# Segmental Pallor and Hair Loss in a Patient With Ross Syndrome: Case Report

**DOI:** 10.1111/1346-8138.70444

**Published:** 2026-08-03

**Authors:** Markus Augusto Martínez Holst, Ana María Sierra Valiente, Francisco Alejandro Silva Navarro, Paola Picazo León, Carolina De La Paz Cruz, Frida Aguilera Villa

**Affiliations:** ^1^ Faculty of Medicine Universidad Anáhuac Mexico Huixquilucan Estado de Mexico Mexico; ^2^ Faculty of Medicine and Health University of New South Wales Sydney NSW Australia

**Keywords:** Anhidrosis, hair loss, hyperhidrosis, pallor, Ross syndrome, tonic pupil

## Abstract

A 30‐year‐old male sought medical consultation due to sudden anisocoria. Clinical examination confirmed Adie's tonic pupil. Subsequently, the presence of segmental anhidrosis with compensatory hyperhidrosis and areflexia established the diagnosis of Ross syndrome. In a 15‐year follow‐up, uncommon manifestations developed in the facial territory affected by compensatory hyperhidrosis: unilateral facial pallor and alopecia following a midline‐defined pattern. This case emphasizes the importance of recognizing neurocutaneous manifestations in dermatology practice and suggests that Ross syndrome may encompass a broader spectrum of dermatological findings than currently recognized.

## 1 Introduction

Ross syndrome (RS) was first described by Alexander Ross in 1958, and since then, fewer than 60 cases have been published in the literature [1]. The syndrome is characterized by the clinical triad of segmental anhidrosis or hypohidrosis, areflexia, and Adie's tonic pupils. Additionally, patients may exhibit compensatory hyperhidrosis in remaining areas of functioning eccrine glands, fatigue, chronic cough, and increased urinary frequency [2]. While the trigger for this rare condition remains unidentified, various case reports have suggested the association of autoantibodies or infectious diseases with the syndrome onset [3]. The underlying mechanism in RS features is believed to be a progressive, selective degeneration of postganglionic autonomic nerves [3]. To support the clinical diagnosis, dilute pilocarpine eyedrops aid in confirming Adie's tonic pupil, while intradermal pilocarpine administration helps identify postganglionic sudomotor dysfunction [3]. Since there is no effective treatment for this condition, management primarily focuses on symptomatic relief.

We report the case of a patient with RS exhibiting classical clinical manifestations and two novel, unreported dermatological features: chronic pallor and hair loss restricted to the facial region affected by compensatory hyperhidrosis. A review of the available literature reveals no prior report of these features. To our knowledge, Mexico has not previously reported a case of RS.

## 2 Case Presentation

A healthy 30‐year‐old male pediatrician with no relevant past medical history sought consultation with an ophthalmologist after noticing sudden onset of anisocoria. On examination, permanent mydriasis of the right eye was noted, with the right pupil unreactive to direct and consensual light stimulation (Figure 1). Bilateral topical ocular instillation of dilute pilocarpine (0.125%) caused marked right pupil constriction (> 0.4 mm) compared to a minimal response in the left unaffected eye at 30 min. Results supported postganglionic damage and the diagnosis of unilateral Adie's tonic pupil. A month later, physical activity revealed hyperhidrosis on the left side of his face. A head CT scan was performed to exclude structural lesions, with no abnormalities noted. Subsequent examination revealed segmental anhidrosis (right face, left trunk and left upper limb) with compensatory hyperhidrosis (left face, right trunk), while sweating in the lower limbs remained intact bilaterally. This distribution was identified by visible asymmetry in sweat distribution during physical exertion, corroborated by consistent patient history. On evaluation of the extremities, 5/5 strength and intact sensation was documented in all four limbs. Nonetheless, the examination of deep tendon reflexes detected areflexia of the left patellar reflex and hyporeflexia of the left ulnar reflex. Findings were consistent with RS. The patient also experienced heat intolerance, and reported that his left arm and hand are “always cold”. He presented one episode of heat stroke with confusion, tachycardia, and loss of consciousness, which resolved with cold therapy in the emergency department. In addition to the classic presentation, approximately 15 years after initial diagnosis, our patient exhibits two unusual dermatological features: segmental pallor across the left forehead and nonscarring alopecia localized to the left scalp region. Both findings were strictly ipsilateral, anatomically concordant, and confined to the facial territory affected by compensatory hyperhidrosis, with gradual onset as reported by the patient. The alopecia presented as diffuse hair thinning with sharp midline demarcation extending from the frontotemporal region to the parietal and vertex areas, most prominent over the left temple where it spanned approximately 6 cm anteroposteriorly. Residual miniaturized hairs were present within the affected area, without skin atrophy, erythema, or scaling (Figure 2). More recently, mild frontotemporal hair thinning was also noted on the right side, though markedly less pronounced. The unilateral pallor was stable and present at rest, and became more pronounced during episodes of profuse sweating (Figure 3), with decreased left facial skin temperature on palpation compared with the contralateral side, even after sweating had ceased. No hair loss or skin color changes were observed beyond the craniofacial region.

**FIGURE 1:**
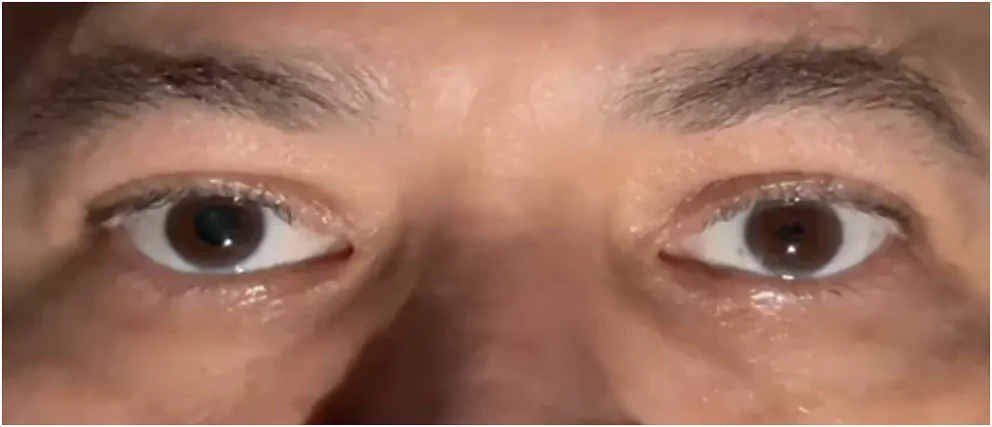
Unilateral right‐sided Adie's tonic pupil: Enlarged right pupil reacting poorly to light, with preservation of normal reactivity in the left pupil.

**FIGURE 2:**
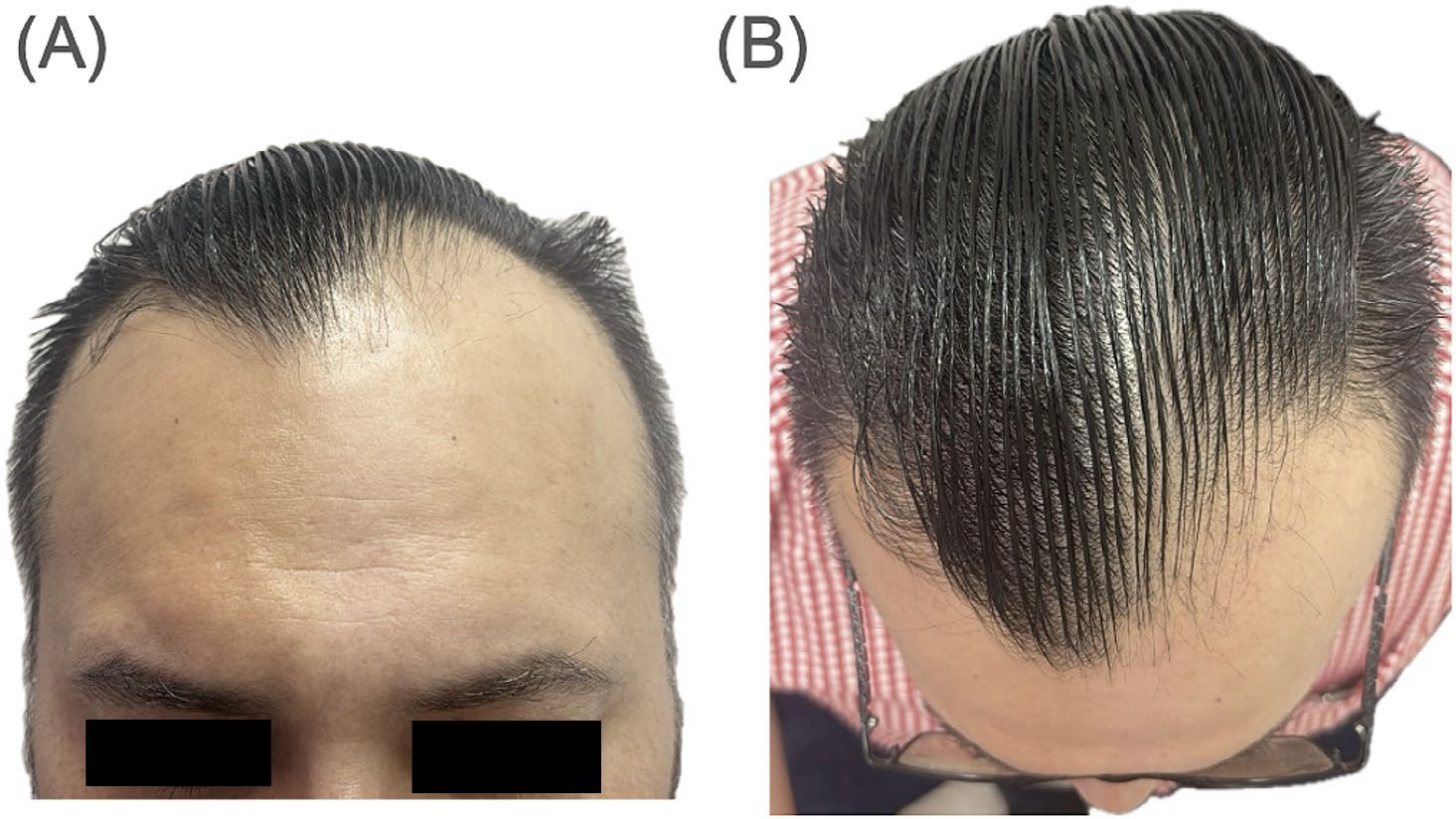
Dermatologic manifestations in a patient with Ross syndrome: Segmental alopecia and facial pallor at rest in hyperhidrotic area (without active sweating); (A) Frontal view showing frontotemporal hairline recession with marked left‐sided involvement, and ipsilateral pallor appreciable across the left hemiforehead. (B) Superior vertex view of left segmental alopecia demonstrating frontotemporal hair thinning with sharp midline demarcation, residual miniaturized hairs within the affected area.

**FIGURE 3:**
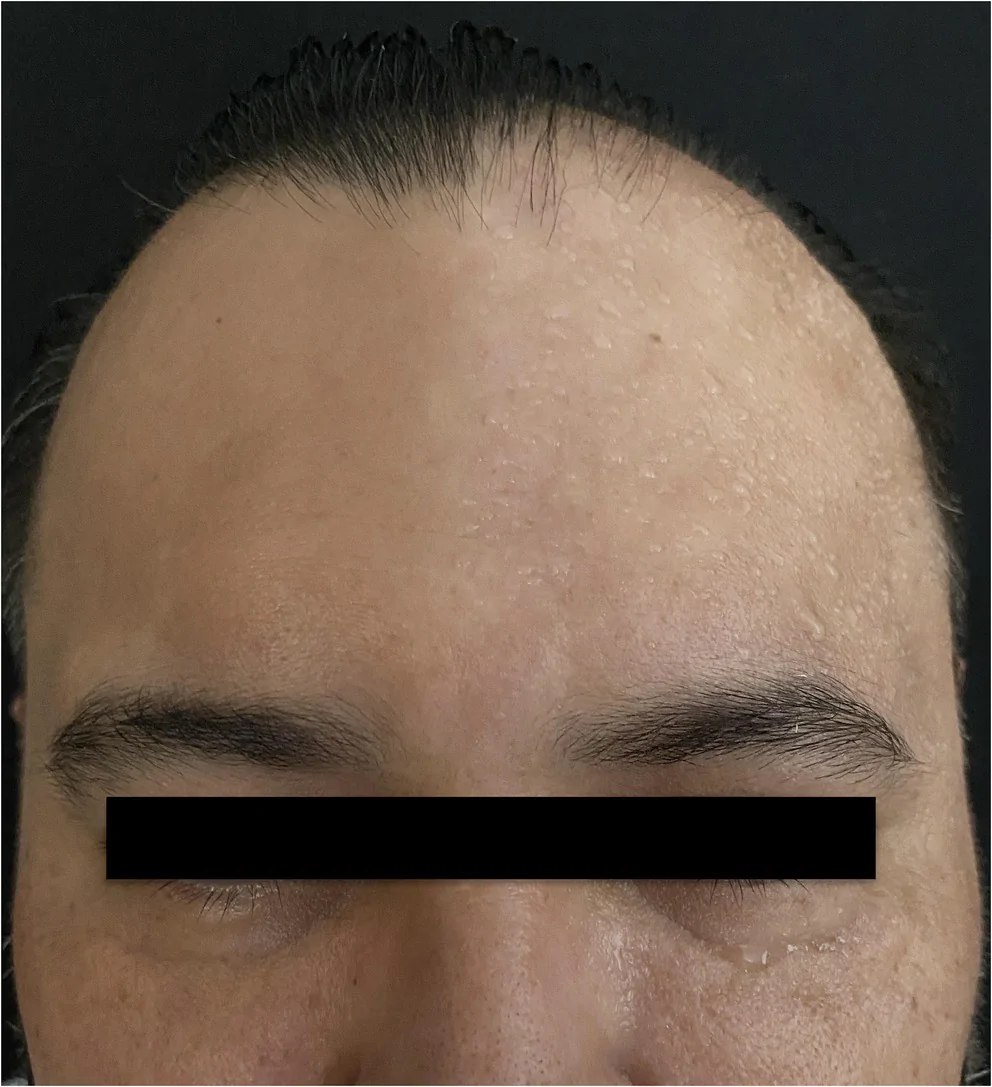
Frontal view of a patient with Ross syndrome during physical exertion, demonstrating the classical distribution of facial segmental anhidrosis (right side) and compensatory hyperhidrosis (left side), alongside the novel finding of paradoxical pallor across the left hemiforehead ipsilateral to the hyperhidrotic area.

## 3 Discussion

The primary cutaneous finding in RS is segmental anhidrosis or hypohidrosis, often accompanied by compensatory hyperhidrosis in unaffected areas. Previously, hyperpigmentation corresponding to hyperhidrotic areas and sparse hairs over the affected anhidrotic segment of the beard and mustache was reported in one case [4]. A detailed skin innervation study in 12 RS patients documented loss of cutaneous blood flow regulation, but no corresponding clinical skin changes were reported [5].

In the present case, the patient exhibited persistent segmental forehead pallor with accentuation during sweating, and alopecia with sharp midline demarcation. Both findings were anatomically concordant with the hyperhidrotic territory and stable across follow‐ups, while hair density and skin color remained unchanged in the contralateral facial anhidrotic segment. In contrast to the classical Harlequin‐type pattern, in which the anhidrotic side remains pale and the hyperhidrotic side tends to be flushed, the pallor in this case was paradoxically confined to the hyperhidrotic segment. To our knowledge, this is the first description of such cutaneous findings in RS.

Nolano et al. found that in normohidrotic skin of RS patients, both sudomotor and vasomotor fibers are markedly reduced yet not completely lost [5]. We speculate that our patient's findings may reflect autonomic dysregulation of vasomotor function coexisting with preserved cholinergic sudomotor activity in these partially denervated territories, resulting in pale hyperhidrotic skin segments.

Given the vascular dependence of hair follicles, one possible explanation is that chronic impairment of cutaneous blood flow in these territories may contribute to the observed localized hair loss [6]. While concomitant androgenetic alopecia (AGA) cannot be excluded in a 45‐year‐old male with modest bitemporal recession, the markedly unilateral hair loss with sharp midline demarcation is unlikely to be explained by AGA alone, as this pattern is inconsistent with the typically bilateral and symmetric distribution of AGA.

This report has limitations. Formal sudomotor testing and histopathological evaluation were not performed due to institutional unavailability, and the proposed pathophysiological mechanism could not be confirmed.

## 4 Conclusion

This case expands the recognized presentation of RS by documenting segmental pallor and hair loss in compensatory hyperhidrotic skin. The possibility of autonomic impairments developing over time reinforces the notion that RS is a progressive disorder whose full clinical spectrum has not yet been characterized. The presence of distinctive cutaneous findings underscores the importance of dermatologists in the diagnostic workup of RS, as skin manifestations may precede or accompany systemic autonomic deterioration.

## Ethics Statement

The authors have nothing to report.

## Consent

Written informed consent was obtained from the patient for publication of this case report and the accompanying images.

## Conflicts of Interest

The authors declare no conflicts of interest.

## Data Availability

Data sharing not applicable to this article as no datasets were generated or analysed during the current study.
